# Sensor Measures of Affective Leaning

**DOI:** 10.3389/fpsyg.2020.00379

**Published:** 2020-04-30

**Authors:** Thomas Martens, Moritz Niemann, Uwe Dick

**Affiliations:** ^1^Medical School Hamburg, Hamburg, Germany; ^2^Institute of Information Systems, Leuphana University, Lüneburg, Germany

**Keywords:** sensor measures, process measures, affect, emotion, motivation, EEG, affective learning, self-regulated learning

## Abstract

The aim of this study was to predict self-report data for self-regulated learning with sensor data. In a longitudinal study multichannel data were collected: self-report data with questionnaires and embedded experience samples as well as sensor data like electrodermal activity (EDA) and electroencephalography (EEG). 100 students from a private university in Germany performed a learning experiment followed by final measures of intrinsic motivation, self-efficacy and gained knowledge. During the learning experiment psychophysiological data like EEG were combined with embedded experience sampling measuring motivational states like affect and interest every 270 s. Results of machine learning models show that consumer grade wearables for EEG and EDA failed to predict embedded experience sampling. EDA failed to predict outcome measures as well. This gap can be explained by some major technical difficulties, especially by lower quality of the electrodes. Nevertheless, an average activation of all EEG bands at T7 (left-hemispheric, lateral) can predict lower intrinsic motivation as outcome measure. This is in line with the personality system interactions (PSI) theory of Julius Kuhl. With more advanced sensor measures it might be possible to track affective learning in an unobtrusive way and support micro-adaptation in a digital learning environment.

## Introduction

That emotion and motivation play a crucial role for all kinds of learning processes is proven in various empirical works, for example the impact of positive emotions (Estrada et al., 1994; Ashby et al., 1999; Isen, 2000; Konradt et al., 2003; Efklides and Petkaki, 2005; Bye et al., 2007; Nadler et al., 2010; Huang, 2011; Um et al., 2012; Plass et al., 2014; Pekrun, 2016). And it is quite difficult to compare the various results because they are built on different theories and different measures. Of course measures, underlying theories and even analytical methods are intertwined with each other forming typical research paradigms. A very prominent research paradigm is self-regulated learning (Pintrich and De Groot, 1990; Zimmerman, 1990; Winne and Hadwin, 1998).

Self-regulated learning emphasizes cognitive and metacognitive processes (e.g., Winne and Hadwin, 1998; Winne, 2018). Even if affective and motivational processes are explicitly mentioned they are reduced to a helping function for the primary cognitive and metacognitive processes (Wolters, 2003; Schwinger et al., 2012). This might be caused by the dominant view of the teacher on learning processes (teaching-learning short circuit – see Holzkamp, 1993; see also Holzkamp, 2015). Moreover, most data gathering techniques are not able to cover affective processes fully because they rely mostly on verbal (self-report) data (see also Veenman, 2011). Because of the holistic nature of emotional processes (Kuhl, 2000a) a verbal report is a simplified representation of emotions.

To lay out a brief theoretical foundation for the process measures used in this study, three major aspects of learning are emphasized here:

1. Learning is always a process over time.

2. Learning is always an internalization process with various degrees. The learning subjects transform themselves for future interaction with the (learning) environment.

3. Affect, emotions, and motivations play a crucial role for learning as an internalization process over time. A higher degree of internalization leads to a number of positive effects: e.g., less perceived effort, higher achievement, more effective use of learning time (Metzger et al., 2012).

Internalization processes go along with positive affect as well as with the dampening of negative affect. Positive affect fosters intuitive learning processes that can be sustained over a long time without any effort (Csikszentmihalyi, 1990). Dampened negative affect supports connecting the inner self as well as self-schemata with the learning topic (as provided by specific learning environments including digital environments) (Kuhl, 2000a, b).

Negative affect as well as the dampening of positive affect stop or at least pause internalization processes of learning. Negative affect usually goes along with analyzing incongruent features of the learning topic that might be threatening (Kuhl, 2000b). Dampening of positive affect freezes the ongoing learning activity and initiates a shift toward more reflective processes of learning like thinking and problem solving (Kuhl, 2000b). So, according to the personality system interactions (PSI) theory (Kuhl, 2000a; Kuhl et al., 2015) it can be assumed that positive and negative affect as well as the dampening of these two are associated with specific processes of self-regulated learning. A sustained negative affect should hinder processes of internalization that could result in processes of intrinsic motivation. Derived from magnetic resonance imaging (MRI) studies negative affect is associated with activities of the left amygdala (Schneider et al., 1995, 1997; Sanchez et al., 2015) and may also result in a higher parietal left-hemispheric activation. There is also some evidence that negative mood is associated with frontal left hemispheric electroencephalographic activity (for an overview see Palmiero and Piccardi, 2017), but empirical results are built on induced emotions and not directly comparable to this study.

Internalization processes initiate processes of deep learning. Especially, resulting knowledge is associated with self-schemata (important aspects of the inner self). The interconnectedness with important aspects of the inner self helps to prevent knowledge from becoming inert (see Renkl et al., 1996). By charging the gained knowledge with personal affect and emotions the recall in various future situations will be much easier. By increasing the chance for recall this will also foster long term memorization, also because every recall in itself is a new association.

Associated with these deep learning processes is the development of a stable interest (e.g., Krapp, 2005). First, often weak associations between learning topics and the inner self could be described as new situated interested (Bernacki and Walkington, 2018). And in the long run as the associations with the inner self become stronger this might lead to stable interest and in the end to an enduring individual interest (see also Hidi and Renninger, 2006).

So far, affective and motivational states within the learning process have been dominantly conceptualized on a meso level time frame, like the postulated impact of positive affect on internalization. Investigations on a micro level time frame like Bosch and D’Mello (2017) will be much more common in the future. Besides methodological challenges how to combine and triangulate data sources from different time levels (see Järvelä et al., 2019), theoretical problems arise. Especially, micro level theories like the cognitive disequilibrium model (D’Mello and Graesser, 2010) has to be interconnected and integrated into higher level models of self-regulated learning (e.g., Winne and Hadwin, 1998, 2008). It can be assumed that the positive effects of affective learning, especially the internalization process cannot be fully supported by digital learning environments. In person-to-person learning situations the teaching person can react to the emotions of the learning person and adapt the learning process accordingly to personal needs. Typically, a person has two main ways for providing affective learning support:

(1) Emotional support, e.g., by soothing someone.

(2) Adaptation of the learning situation, e.g., by providing individualized feedback.

So far, direct emotional social support must be provided by a human being. So we will explore how a digital learning environment can be adapted to individual needs that change during the learning process. Micro-Adaptation (for an overview see Park and Lee, 2003) is working on the premise that interactions between learner and learning environment lead to adaptation. The learner provides a “signal” and the learning environment reacts with a specific adaptation. Whereas the actions of teachers might be intuitive and to some degree undefined, the algorithms of a learning environments must be exactly defined. At first, motivational or emotional states must be measured and identified subsequently. Secondly, adaptive reactions to these identified states must be defined.

For the purpose of micro adaptation in a learning environment it is important to gather information during the learning process (Panadero et al., 2016). A simple way for doing so is embedded experience sampling (Larson and Csikszentmihalyi, 1983; Csikszentmihalyi and Larson, 1987; Hektner et al., 2007). Embedded Experience sampling is usually based on short questionnaires that will be presented in defined time intervals or event related. Clearly, embedded experience sampling is able to track the process character of learning. But two pitfalls will remain: these are still self-reports who will only reflect emotional and motivational states that can be verbally expressed. In this way, rather unconscious processes cannot be reported (at least for a part of people who cannot access their feelings easily). In sum, embedded experience sampling can only convey the verbally expressed motivations and emotions which reflect cognitive thoughts rather than pure motivations or emotions. The second pitfall is that embedded experience sampling as a specific form of self-report will always disturb the learning process. Therefore, additional measures are required that can unobtrusively measure processes of affective learning.

## Research Questions

So, in this study we want to measure processes of affective learning unobtrusively with physiological data. Two types of data will be used for prediction: electrodermal activity (EDA) and electroencephalography (EEG). Two types of predicted data will be reported: online or process measures (experience sampling) and outcome measures for self-regulated learning.

## Materials and Methods

### Participants

Subjects participated in an 1-h long learning experiment. Learning material was taken from a course in a higher semester. Individuals who had already attended these courses were excluded from participating in the study.

Data were gathered in two cohorts (see Table 1). The first cohort consisted of 65 students of Psychology and was tested between October 2016 and February 2017. One subject pulled out of the study due to self-reported headache caused by the EEG headset, reducing the number of participants in the first cohort to 64 (*n* = 14 male). Subjects in the first cohort were between 19 and 38 years of age (*M* = 22.59, *SD* = 3.23). The second cohort consisted of 36 students of Psychology (*n* = 13 male). Here, data were collected in February and March of 2018 and age ranged between 18 and 32 (*M* = 22.14, *SD* = 3.66). The experiment was identical in both cohorts. Cohorts only differed in the wearable devices employed for data collection. In the first cohort, 31 subjects used the Emotiv Insight EEG headset, and 32 the InterAxon Muse EEG headset. Data of the Muse headset had to be discarded due to technical problems with data collection. Data were collected on smartphones (companion devices) with the use of Apps programmed specifically for the experiment. Technical problems with the Muse headset were only observed using a third generation Motorola Moto G running Android 6.0.1. There were no issues when using an alternative device (LG Nexus 5× running Android 6.0.1), nor with the Emotiv Insight and any companion device. The second cohort was scheduled to compensate for the lost data. In the second cohort only the Emotiv Insight headset was used. 24 complete EEG datasets exist for the first cohort, and 30 for the second. In addition to the headsets, the wrist-worn wearable device AngelSensor was used in the first cohort. It was worn by the subjects on the dominant hand (i.e., right hand for right-handed individuals). Data were discarded due to problems with handling the device. In both cohorts, subjects wore the Microsoft Band 2 (MSB2) on their non-dominant hand (i.e., left hand for right-handed individuals). 47 complete MSB2 datasets exist for the first cohort, and 26 for the second. Complete datasets for both EEG and MSB2 exist for 21 subjects in the first cohort, and 22 in the second.

**TABLE 1:** Sample sizes.

	*n*	EEG (Emotiv Insight)	EDA (MSB2)	EEG ∩ EDA
Cohort I	64	24	47	21
Cohort II	36	30	26	22
Σ	100	54	73	43

### Procedure

The experiment took place in a soundproof experimental booth. Before the learning experiment, subjects were asked to put on the wearables themselves. Good fit was ensured by the test supervisor. They were then given the experimental instruction. After the first set of questionnaires, the subjects were shown a demo item of the parsimonious questionnaire to familiarize them with the scales. The subjects were then handed the learning material and the learning session started. During these 60 min of learning, participants were interrupted every 4.5 min with a vibration alarm, and asked to fill out the parsimonious questionnaire concerning their motivational state on a smartphone. Sensor data from the wearable devices were collected throughout the learning session. The learning session was terminated after 60 min and the subjects filled out the second set of questionnaires including the Multiple-Choice-Test. Retrospective questionnaires were presented before the Multiple Choice-Test.

### Learning Material

Participants were given study material from a higher semester of educational psychology. The material consisted of a nine-page excerpt about intrinsic and extrinsic motivation from a German textbook on pedagogical psychology (Schiefele and Köller, 1998), as well as two case studies. Each case study describes a university student with motivational struggles. Subjects were asked to explain the problems using the theory provided in the textbook excerpt, and to make recommendations regarding possible courses of action.

### Process Measures (Experience Sampling)

**FIGURE 1:**
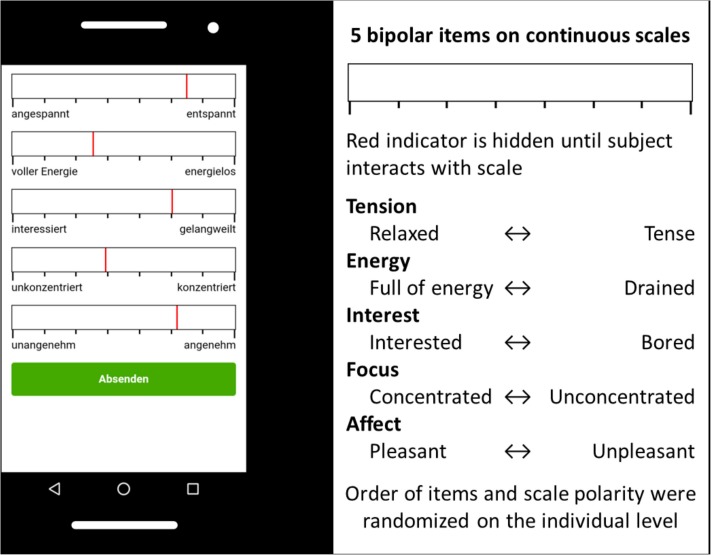
Experience sampling with a parsimonious questionnaire.

To assess subjects’ affective states during the learning task, a parsimonious questionnaire was devised and implemented (see Figure 1). It was presented on a smartphone, and subjects used their fingers as input on the touchscreen to complete it. Subjects were alerted to fill out the questionnaire every 270 s (4.5 min) via a vibration alarm lasting 1 s, for a total of 13 experience samples. To our knowledge, no recommendations exist for determining the frequency of such a high-frequency experience sampling. The 270 s were therefore determined in informal pre-tests to be the minimum amount of time before the experience sampling was perceived as annoying and intrusive. The instruction asked participants to state how they were feeling prior to being interrupted by the vibration alarm. Answers had to be given within 60 s to be processed as valid data The questionnaire was designed to be completed in as little time as possible. Responding time averaged 16.36 s (*SD* = 7.36, Median = 14.64), meaning participants spent about 4 min of the 60-min learning session answering the questionnaire (6%).

The questionnaire consists of five bipolar sliding scales (sliders) ranging between two endpoints marked with affective words (end items). Sliding scales differ from rating scales (e.g., Likert scales) in that subjects are free to choose any value between the arbitrarily set end values of −4000 to +4000. The scales are marked with eight tick marks to provide orientation to the subjects, visually mimicking an eight-point Likert scale. Sliders have been shown to yield comparable results to ordinary categorical response formats in online surveys (Roster et al., 2015). The five bipolar sliding scales used are interest, energy, valence, focus, and tension. They range between the end items bored – interested [gelangweilt – interessiert] (interest), without energy – full of energy [energielos – voller Energie] (energy), unpleasant – pleasant [unangenehm – angenehm] (valence), focused – not focused [unkonzentriert – konzentriert] (concentration), and relaxed – tense [entspannt – angespannt] (tension). Similar or identical items have been used in the past to assess affective states in other longitudinal designs (Triemer and Rau, 2001; Wilhelm and Schoebi, 2007). Items were chosen to ensure a short response time (resulting in 16 s response time in average). Intervals between measurements are typically measured in hours, while in our current study we used a much higher measurement frequency of only minutes (high-frequency experience sampling). Order and polarity of the scales were fully randomized, but kept consistent for each subject. On each experience sample, the indicators on the sliders were hidden until subjects first interacted with the scale. Subjects were therefore not able to see which point on the continuum they had previously selected.

### Outcome Measures

Prior to the learning session, subjects gave their demographic data. At the end of the learning session, learning outcome was measured with a multiple choice test, and subjects gave retrospective self-reports regarding their motivational and affective states across the whole learning session. Subjects filled out part I and II of Dundee Stress State Questionnaire (DSSQ Matthews et al., 1999). Part I is the Mood and Affect portion and is equivalent to the UWIST Mood Adjective Checklist (Matthews et al., 1990). It consists of 29 affective adjectives on the four subscales Energetic Arousal, Tense Arousal, Hedonic Tone, and Anger/Frustration. Subjects are asked to state to which degree they felt the given affective state over the course of the learning session. Part II of the DSSQ concerns motivation and consists of the Intrinsic Motivation and Workload subscales, the latter of which is the NASA-TLX questionnaire in modified form (Hart and Staveland, 1988). In addition, we presented a more finely grained measure of retrospective regulation derived from Self-Determination Theory (Ryan and Decy, 2000). It distinguishes between Amotivation, External Motivation, Introjected Motivation, Identified Motivation, Intrinsic Motivation, and Interest (Prenzel and Drechsel, 1996). Additional questionnaires that measure the Integrated Model of Learning and Action (Martens, 2012) will not be reported in this article.

### Electrodermal Activity

Microsoft Band 2 (MSB2) was used to collect Skin Resistance measurements at the wrist of the subjects’ off-hand. Galvanic Skin Resistance (GSR) is the inverse of Skin Conductance and a measure of EDA. The MSB2 samples GSR at 5 Hz. Electrode contact was ensured by tightening the strap of the MSB2 around the subjects’ wrists. No gel was used.

### Electroencephalography

**FIGURE 2:**
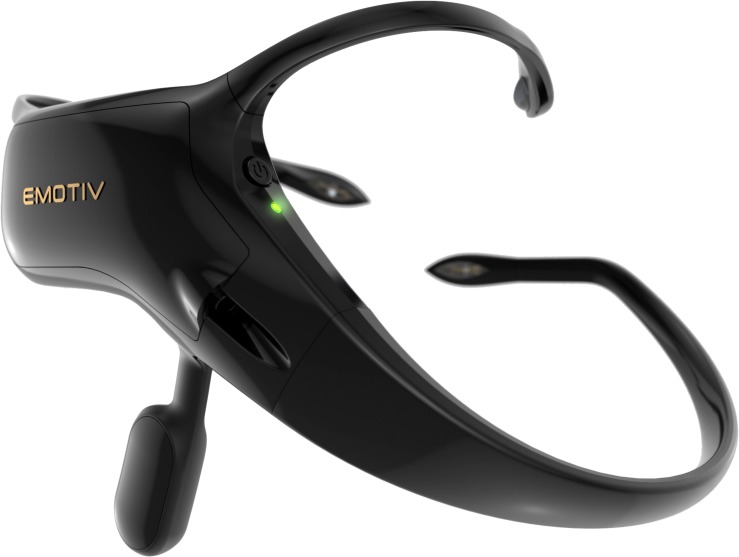
The asymmetric Emotiv Insight Headset. Reproduced with permission.

Wireless, wearable Headsets were used to collect EEG measures. Approximately half of the first cohort tested donned the Emotiv Insight (see Figure 2) and the other half the InterAxon Muse EEG Headsets. Data of the Muse Headset had to be discarded due to problems with data collection. We opted to test a second cohort using the Emotiv Insight to systematically increase the sample size.

The Emotiv Insight uses five dry electrodes to measure EEG on the scalp. The electrode positions are roughly equivalent to the standardized electrode positions AF3, AF4, T7, T8, and Pz according to the modified combinatorial nomenclature (MCN). The Emotiv Insight is an asymmetrical headset with the electronics, battery, and reference electrodes on the left side of the device. It is fixated over and behind the left ear, where the T7 electrode and two reference electrodes make firm contact with the head. The Emotiv Insight uses two common mode sense (CMS)/driven right leg (DRL) reference electrodes on left mastoid process. The remaining electrodes are attached to non-adjustable plastic arms that wrap around the skull. The headset sits tight on the head, although positions of the remaining four electrodes vary somewhat from subject to subject.

The Emotiv Insight does not expose the raw EEG data stream out-of-the-box, although licensing options exist. By default, the Emotiv Insight returns precomputed power values for the theta (4–8 Hz), alpha (8–12 Hz), lower beta (12–16 Hz), upper beta (16–25 Hz), and gamma (25–45 Hz) bands for each of the five electrode positions. According to the FAQ^1^, the Insight samples at 2048 Hz, which is then downsampled to 128 Hz. Documentation about probable additional filtering is non-existent. Band power data are computed via Fast-Fourier-Transformation and returned at 8 Hz, employing a 2 s Hanning window with a step size of 125 samples.

### Data Analysis

Complete datasets for EEG and EDA combined existed for 45 subjects, we therefore opted to attempt prediction using EDA and EEG data separately. This maximizes predictive power for each sensor measure, while disallowing direct comparisons of predictive power between the sensor measures.

Reports of statistical analysis and results are split in two. First, we attempt to predict all 13 experience samples on each of the 5 scales employed. Here, data from the 270 s preceding each experience sample were used. From this time interval, physiological data where subjects were busy answering the parsimonious questionnaire were removed. With this procedure approximately 6% of the data were discarded. The resulting time varies from person to person and from sample to sample, the metrics we computed and explained below are therefore based on varying amounts of data. Secondly, we attempt to predict the data gathered from retrospective questionnaires. For this, we used sensor data gathered across the whole learning session, minus times when subjects were busy answering the parsimonious questionnaire.

We estimate predictive potential of measured sensor data by using and evaluating two machine learning regression algorithms. All complete datasets were included in the analysis.

### Preparation of Physiological Data

Each sensor outputs a sequence of sensor data for proband *i* during an experiment. In order to predict questionnaire values, the raw data measured by each sensor are transformed to a set of features that describe the sensor data sequence. Features for the machine learning process are generated by splitting sensor data into 13 segments corresponding to the intervals for experience sampling. The following features were generated for EDA: mean, median, standard deviation, maximum, minimum, difference between maximum and minimum value, difference between medians of first 30 s and last 30 s (denoted tendency in the evaluation). The same features as for EDA were calculated for the EEG for each electrode position and precomputed power band (theta, alpha, low beta, high beta, gamma). In addition, we computed several indices of brain activity: low beta divided by alpha for all sites (denoted BLA), beta divided by the sum of theta and alpha (denoted NASA). Furthermore, anterior and temporal laterality indices were used comparing activity at left hemispheric sites across all bands with activity at right hemispheric sites across all bands. Lateral T is computed as the difference between T7 and T8 for all frequency bands and Lateral AF as the difference between AF3 and AF4.

### Machine Learning

Two different machine learning models were employed to evaluate predictive potential of sensor data, one linear (Ridge Regression) and one non-linear model (Gradient Boosting with the XGBoost algorithm). Training the respective models is done by a model-specific training procedure that adjusts the parameters of the model to training data and an evaluation procedure that predicts target values for a set of features. The machine learning models learn functions that map sets of features *x*_*i*_ to questionnaire values *y*_*i*_ by minimizing a loss function that depends on the machine learning model. The models are trained on training data that consist of feature-label pairs (*x*_*i*_, *y*_*i*_) of probands.

Ridge Regression (Hoerl and Kennard, 1970) is a l2-regularized linear regression model. The regularization parameter penalizes large weights of the model. Gradient Boosting (Friedman, 2001) is a more complex non-linear model that has shown impressive results on a large variety of regression and classification problems (Chen and Guestrin, 2016). The model uses several hyperparameters to control the complexity of the learned model. In our experiments we use the XGBoost algorithm of Chen and Guestrin (2016).

The data structure determines the validation procedure. For the purpose of cross-validation one data point is systematically left out. For predicting a single outcome measure the leave-on-out (LOO) cross-validation method was used. For predicting the 13 data points nested within an individual during the learning experiment the leave-one-proband-out (LOPO) cross-validation method was used. Both forms of cross-validation are very similar and iteratively assign a part of the data set to be validation data that cannot be used for training, but they differ in regard to the underlying data structure. Especially, the subsequent analytical steps are similar for both procedures.

The LOPO cross-validation simulates a prediction based on observed sensor data of previously unseen probands. To this end, let *X*_*i*_ = {(*x*_*i*_^1^, *y*_*i*_^1^), …, (*x*_*i*_^13^, *y*_*i*_^13^)} be a set of 13 feature-label pairs of process outcomes of proband *i*. Let *D* = {*X*_1_, *X*_2_, …, *X*_*n*_} be the set of all those feature-label sets of probands *I* = {1, 2, …, *n*}. We iteratively choose proband *i* = 1, …, *n* and remove its data subset *X*_*i*_ from the pool of training data *D*. The resulting data set *D*_–i_ is used to train the machine learning regressor which is then evaluated on the remaining set *X*_*i*_ using any of the error functions of the last paragraph. The final outcome of LOPO-CV is computed by averaging over all probands.

The LOO cross-validation instead only removes a single feature-label pair. Let *D* = {(*x*_1_, *y*_1_), (*x*_2_, *y*_2_), …, (*x*_*n*_, *y*_*n*_)} be the set of all feature-label pairs corresponding to probands *I* = {1, 2, …, *n*}. We iteratively choose proband *i* = 1, …, *n* and remove its feature-label pair (*x*_*i*_, *y*_*i*_) from the pool of training data *D*. The resulting data set *D*_–i_ is again used to train the machine learning regressor which is then evaluated on the remaining pair (*x*_*i*_, *yi*) using any of the error functions of the last paragraph. Both Ridge Regression and XGBoost use hyperparameters that help to avoid overfitting by adjusting the complexity of the learned model. Parameters are tuned on each LOPO or LOO training set *D*_–_*_*i*_* separately. The hyperopt-library (Bergstra et al., 2013) was used to perform parameter tuning with a threefold cross-validation on *D*_–_*_*i*_*.

Features were selected on each LOPO or LOO training set *D*_–_*_*i*_* separately, whenever stated. The recursive feature elimination with cross-validation (RFECV) algorithm (Guyon et al., 2002) was used.

For evaluating predictions two error functions were used, namely root mean square error (RMSE) and mean absolute error (MAE). Additionally, Pearson correlation coefficients between predictions and real values were computed.

The baseline method for questionnaires predicts the questionnaire values of proband *i* to be the mean value of questionnaire values of all other probands. That is, ${\hat{y}}_{i}^{b\,l}=\text{mean}\,\left(\left\{y_{1},\ldots ,y_{n}\right\}\backslash \left\{y_{i}\right\}\right)$ where \ denotes the relative complement of sets. This baseline does not take into account any sensor data but serves as a sensibility check for results achieved by the machine learning models. Analogously, the baseline method for experience sampling predicts the values for each sample as ${\hat{y}}_{i}^{b\,l,1}={\hat{y}}_{i}^{b\,l,2}=\ldots ={\hat{y}}_{i}^{b\,l,13}=\text{mean}\,\left(\left\{y_{1}^{1},y_{1}^{2},\ldots ,y_{1}^{13},y_{2}^{1},\ldots ,y_{n}^{13}\right\}\backslash \left\{y_{i}^{1},\ldots ,y_{i}^{13}\right\}\right)$. Meaningful predictions have to be better than the baseline method. In this way, general trends over time that are shared by all probands cannot be predicted significantly by the machine learning model.

## Results

Prediction results are presented compared to the corresponding baseline measure. Evaluation errors are compared to the baseline for significance using student’s *t*-tests.

### Prediction of Experience Sampling Using Electrodermal Activity (EDA)

No machine learning model was able to predict process measures from experience sampling significantly above baseline using median EDA (see Table 2).

**TABLE 2:** Results for predicting experience sampling using electrodermal activity (EDA).

	RMSE	MAE	*r*	*p*	*d*
**Interest**					
Ridge	1645.2	1324.3+	0.003		
Boosting	1600.3+^∗^	1291.2+^∗^	0.171	0.092	0.20
Baseline	1636.9	1327.4	−0.813		
**Energy**					
Ridge	1590.0+	1292.4+	0.046		
Boosting	1586.9+	1299.6+	0.061		
Baseline	1596.0	1302.3	−0.815		
**Focus**					
Ridge	1630.0+	1339.4+	0.032		
Boosting	1610.8+	1320.3+	0.132	0.143	0.17
Baseline	1633.6	1347.5	−0.747		
**Valence**					
Ridge	1573.8	1280.3	−0.828		
Boosting	1577.6	1284.5	−0.146		
Baseline	1573.7	1280.3	−0.828		
**Tension**					
Ridge	1660.8	1372.6	−0.527		
Boosting	1659.5	1370.6	−0.675		
Baseline	1654.7	1367.2	−0.791		

*^+^improved performance over baseline. ^∗^denotes significant improvement.*

### Prediction of Experience Sampling Using Electroencephalography (EEG)

The machine learning model was not able to predict process measures from experience sampling significantly above baseline using features of the Emotiv Insight (see Table 3).

**TABLE 3:** Results for predicting experience sampling using electroencephalography (EEG).

	RMSE	MAE	*r*
**Interest**			
Ridge	1867.9	1529.1	−0.355
Boosting	1779.2	1427.8+	−0.061
Baseline	1761.9	1438.2	−0.835
**Energy**			
Ridge	1852.2	1545.0	−0.498
Boosting	1774.6	1469.8	−0.201
Baseline	1715.7	1433.8	−0.831
**Focus**			
Ridge	1818.0	1485.0	−0.018
Boosting	1850.3	1527.9	−0.166
Baseline	1783.9	1469.7	−0.791
**Valence**			
Ridge	1736.9	1401.5	−0.215
Boosting	1689.7	1365.3	−0.131
Baseline	1654.1	1340.2	−0.786
**Tension**			
Ridge	1738.5	1379.4	0.039
Boosting	1715.1	1389.9	−0.080
Baseline	1650.3	1341.6	−0.791

*+: improved performance over baseline.*

### Prediction of Outcome Measures Using Electroencephalography (EEG)

Out of the 12 outcome measures we employed, we were able to predict Intrinsic Motivation as measured by the DSSQ significantly above baseline using sensor data from the Emotiv Insight (see Table 4).

**TABLE 4:** Predicting outcome measures with EEG using leave-one-out cross validation (LOO-CV).

	REL	RMSE	MAE	*r*	*p*	*d*
**Amotivation**	0,68					
Ridge		0.535+	0.400+	−0.076		
Boosting		0.557	0.412	−0.099		
Baseline		0.538	0.408	−1.000		
**External motivation**	0,61					
Ridge		0.509	0.425	−0.066		
Boosting		0.529	0.408	−0.515		
Baseline		0.491	0.382	−1.000		
**Introjected motivation**	0,38					
Ridge		0.869	0.756	−0.207		
Boosting		0.789	0.649	−0.414		
Baseline		0.737	0.618	−1.000		
**Identified motivation**	0,60					
Ridge		0.960	0.757	−0.406		
Boosting		0.863	0.693	−0.468		
Baseline		0.801	0.642	−1.000		
**Intrinsic motivation**	0,69					
Ridge		0.804	0.653	−0.376		
Boosting		0.734+	0.609+	−0.119		
Baseline		0.739	0.610	−1.000		
**Interest**	0,80					
Ridge		0.985	0.857	−0.223		
Boosting		0.940	0.816	−0.409		
Baseline		0.838	0.701	−1.000		
**DSSQ II intrinsic motivation**	0,81					
Ridge		1.079+^∗^	0.815+^∗^	0.685	0.004	0.58
Boosting		1.406+^∗^	1.115+^∗^	0.335	0.069	0.35
Baseline		1.516	1.310	−1.000		
**DSSQ II workload**	0,37					
Ridge		1.236	0.990	−0.665		
Boosting		1.151 +	0.937+	0.290		
Baseline		1.231	0.979	−1.000		
**DSSQ I tense arousal**	0,89					
Ridge		0.623	0.517	−0.519		
Boosting		0.644	0.549	−0.318		
Baseline		0.621	0.512	−1.000		
**DSSQ I anger/frustration**	0,87					
Ridge		0.611	0.498	−0.591		
Boosting		0.591+	0.468+	0.144		
Baseline		0.610	0.492	−1.000		
**DSSQ I energetic arousal**	0,90					
Ridge		0.808	0.695	−0.252		
Boosting		0.753	0.628+	0.033		
Baseline		0.729	0.639	−1.000		
**DSSQ I hedonic tone**	0,88					
Ridge		0.597+	0.489	−0.366		
Boosting		0.611	0.492	0.133		
Baseline		0.598	0.486	−1.000		

*^+^improved performance over baseline. ^∗^denotes significant improvement.*

**FIGURE 3:**
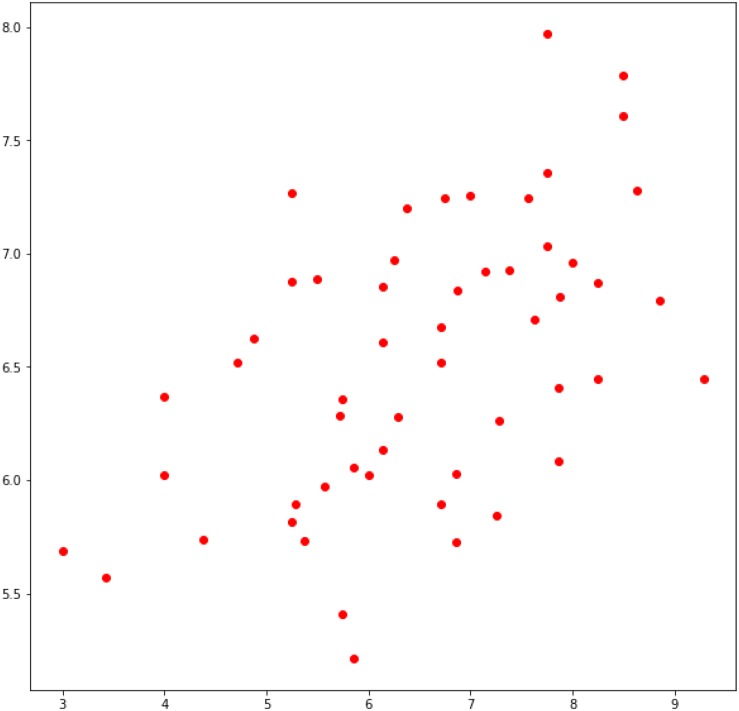
Plot of real values for Intrinsic Motivation (*x*-axis) vs. predicted values via LOO procedure (*y*-axis).

Table 4 shows the average prediction results with LOO cross-validation for outcome measures based on EEG sensor features. We compare predictive performance of ridge regression, XGBoost and the baseline method using RMSE and MAE as well as label-prediction correlation (LPC). As addional information we added the Reliability (REL) of the scales estimated with Cronbach’s Alpha. Both ridge regressions as well as XGBoost significantly outperform the baseline method using a student’s *t*-test with *p*-values of 0.004 and 0.069. The effect sizes are d = 0.58 and d = 0.35, resp. Label-prediction correlation is also visualized by Figure 3 that plots real values of intrinsic motivation for all probands (*x*-axis) in comparison to the predicted values of XGBoost (*y*-axis).

In Table 5 we show the features with highest average weights as learned by XGBoost and averaged over all cross-validation iterations. We like to note that the prediction performance can be a misleading quantity as XGBoost is a non-linear regression method that predicts based on non-linear combinations of features. Consequently, a high importance of a feature does not entail that the feature has large predictive power on its own. For comparison, the highest Pearson correlation coefficients between features and intrinsic motivation are listed in Table 6. If we only consider medians, Table 7 shows the features predicting intrinsic motivation with highest correlations.

**TABLE 5:** Most important features according to the average weights of XGBoost.

Feature	Weight
T7 Nasa max	0.20
T7 BETA_LOW median	0.10
Pz BETA_HIGH tendency	0.10
T Lateral ALPHA maxmin	0.09
T7 BLA min	0.09
T8 BETA_LOW max	0.06
Pz GAMMA tendency	0.05
T8 BETA_HIGH mean	0.05
AF Lateral ALPHA min	0.03
T7 BETA_LOW mean	0.03
T7 THETA min	0.03

**TABLE 6:** Features with highest absolute Pearson correlation coefficients with intrinsic motivation.

Feature	Weight
T7 Nasa max	−0.53
T7 GAMMA median	−0.44
T7 BETA_HIGH median	−0.42
T7 THETA median	−0.38
T7 Nasa maxmin	−0.37
T Lateral THETA tendency	−0.36
T7 ALPHA median	−0.35
T8 BETA_LOW std	−0.35
T7 BETA_LOW std	−0.35
T7 BETA_LOW median	−0.34

**TABLE 7:** Features with highest correlation with Intrinsic Motivation considering medians.

Feature	Weight
T7_GAMMA_median	−0.44
T7_BETA_HIGH_median	−0.42
T7_THETA_median	−0.38
T7_ALPHA_median	−0.35
T7_BETA_LOW_median	−0.34
nasa_AF4_median	−0.26
T8_GAMMA_median	−0.26
lateral_T_BETA_HIGH_median	−0.25
AF4_BETA_HIGH_median	−0.24
T8_BETA_HIGH_median	−0.24

## Discussion

The effect that an average activation of all EEG bands at T7 (left-hemispheric, lateral) can predict lower intrinsic motivation as outcome measure after the learning effect is in line with assumptions of the PSI theory (Kuhl, 2000b, 2001). Kuhl (2000a, 2001) predicts that activating left hemispheric macro systems – especially object recognition – will inhibit right hemispheric macro systems – especially the extension memory. It can be derived that processes of intrinsic motivation need active right hemispheric activation. The extension memory is the bridge to all self-experiences and self-schemata and therefore a key system for internalization processes proposed by self-determination theory (Ryan and Decy, 2000). Nevertheless, the data presented here must be interpreted very carefully. The direct measurement of right hemispheric activation could not be achieved in this study. This might be due asymmetric design of the head set: the delicate placing of the right electrode opposed to the tight grip of left electrode.

In this work consumer grade wearables for EEG and EDA with the selected features failed to predict emotions measured with short questionnaires (embedded experience sampling) that were repeatedly presented during the learning experiment. This gap can be explained by some major technical difficulties:

1. The grip of the consumer grade EEG is asymmetrical and not as tight as a professional EEG set. In addition, no liquids were used in the experiment to foster the electric flow. This argument can be repeated for the consumer grade measurement of EDA. The wrist band guarantees no tight pressure to the skin and was not supported by additional liquids.
2. Internal programs of the consumer grade electronics were not fully disclosed, so compression algorithms may have spoiled the data to some extent.
3. The general setting of this natural learning experiment might not invoke enough measurable arousal and especially not galvanic skin response. The learning situation used in this experiment was intentionally quite common for university student.
4. It cannot be fully excluded that embedded experience sampling might not measure the same processes as the EEG or the EDA. Experience sampling is still a form of verbal expression that reflects emotions. But of course, this expression might be distorted by the very same self-reflecting processes.

The fourth argument can – to some degree – be defused by the fact that this work can predict self-report data for the outcome variable intrinsic motivation.

## Outlook

Some of the major flaws in this study will be healed in the following study by using professional equipment for EDA and EEG. Furthermore, emotions will be measured by facial expressions. The authors still believe that unobtrusive measures of affective learning are very important for understanding learning processes. Subsequently, a theoretical and methodological coevolution will be needed that covers learning processes on micro as well as meso level and integrates affective and motivational regulation processes more deeply into theories of self-regulated learning. This will hopefully be the basis for successfully adapting digital learning environments.

## Data Availability Statement

The datasets generated for this study will not be made publicly available to maintain participants’ confidentiality. Requests to access the datasets should be directed to the corresponding author.

## Ethics Statement

Ethical review and approval was not required for the study on human participants in accordance with the local legislation and institutional requirements. The patients/participants provided their written informed consent to participate in this study.

## Author Contributions

TM and MN designed the experiments. TM, MN, and UD wrote the manuscript. MN executed the experiments. TM, MN, and UD analyzed the data.

## Conflict of Interest

The authors declare that the research was conducted in the absence of any commercial or financial relationships that could be construed as a potential conflict of interest.

## Footnotes

1 https://www.emotiv.com/knowledge-base/what-is-the-sampling-rate-for-the-emotiv-insight-and-why-has-it-been-designed-this-way/
